# Translation and validation of the Thai clinical frailty scale and classification tree in older adults

**DOI:** 10.1186/s12877-025-06013-5

**Published:** 2025-05-14

**Authors:** Panas Jesadaporn, Siripong Teepaneeteerakul, Nuttanun Wongsarikan, Kochaphan Phirom, Supannika Poonthananiwatkul, Panita Limpawattana

**Affiliations:** 1 Division of Geriatrics, Department of Internal Medicine, Faculty of Medicine, Chiang Mai University, Chiang Mai, 50200 Thailand; 2 Research Unit of Medicine, Department of Internal Medicine, Faculty of Medicine, Chiang Mai University, Chiang Mai, 50200 Thailand; 3 Research Institute for Health Sciences, Chiang Mai University, Chiang Mai, 50200 Thailand; 4 Quality Center, Bangkok Hospital Phuket, Phuket, 83000 Thailand; 5 Division of Geriatric Medicine, Department of Medicine, Faculty of Medicine, Khon Kaen University, Khon Kaen, 40002 Thailand

**Keywords:** Frailty, Geriatric assessment, Validation study, Reproducibility of results, Thailand

## Abstract

**Background:**

The Clinical Frailty Scale (CFS) is widely used for frailty assessment, but has not yet been formally validated for use in Thai populations. This study evaluated the reliability and validity of the Thai versions of the CFS (CFS-Thai) and its Classification Tree (CFS-CT-Thai).

**Methods:**

In this cross-sectional study, 213 participants aged ≥ 65 years (127 outpatients and 86 inpatients) were enrolled from two tertiary care hospitals in Thailand. The CFS and CFS-CT were translated into Thai using standard procedures. Inter-rater reliability was evaluated in a subsample of 53 inpatients. Concurrent validity was examined using the Thai version of the FRAIL scale (T-FRAIL), the Eastern Cooperative Oncology Group Performance Status (ECOG PS), and the modified Thai Frailty Index (mTFI).

**Results:**

The CFS-Thai showed strong inter-rater reliability (κ = 0.80, *p* < 0.001) and excellent agreement with the CFS-CT-Thai (κ = 0.94, *p* < 0.001). It demonstrated moderate correlation with T-FRAIL (ρ = 0.53) and strong correlation with ECOG PS (ρ = 0.76) and mTFI (ρ = 0.73). Using mTFI as the reference standard, the CFS-Thai showed high sensitivity (92.7%) and lower specificity (55.0%) at cut-off ≥ 4 (AUC = 0.74, 95% CI: 0.62–0.86), while cut-off ≥ 5 improved specificity (79.3%) and retained high sensitivity (93.5%) (AUC = 0.86, 95% CI: 0.81–0.92). ECOG PS ≥ 2 provided balanced diagnostic performance (sensitivity 83.9%, specificity 93.3%, AUC = 0.89, 95% CI: 0.82–0.95).

**Conclusions:**

The CFS-Thai and CFS-CT-Thai are reliable and valid instruments for frailty assessment in Thai older adults. Their diagnostic accuracy supports integration into clinical practice, especially in settings with limited geriatric expertise. Further studies should examine their implementation across diverse populations and their predictive value for clinical outcomes.

## Introduction

Frailty is a clinical syndrome characterized by reduced physiological reserves across multiple systems, leading to diminished resilience to stressors and increased vulnerability to adverse health outcomes in older adults [1–3]. A recent systematic review and meta-analysis reported a significant burden of frailty in Southeast Asia. The pooled prevalence among community-dwelling older adults in this region was 11.3% (95% CI: 8.5–14.5%), with country-specific estimates ranging from 5.7% in Singapore to 21.7% in Vietnam [4]. In Thailand, previous studies have reported frailty prevalence between 8.7% and 22.0% [4]. The observed variability has underscored the importance of timely identification and prompted the development and validation of a range of frailty assessment tools.

Early detection of frailty enables the identification of modifiable risk factors and supports targeted interventions [5–8]. However, the lack of a universally accepted gold standard for frailty assessment has resulted in the use of diverse tools with varying conceptual frameworks and performance characteristics. These range from rapid screening instruments to more comprehensive clinical evaluations [9, 10].

International clinical practice guidelines from the International Conference of Frailty and Sarcopenia Research (ICFSR) and the Asia-Pacific region endorse validated tools such as the Frailty Phenotype (FP) and the FRAIL scale for the early identification and management of frailty in older adults [5, 7, 11, 12].

The FP assesses frailty through five criteria: unintentional weight loss, exhaustion, reduced grip strength, slower walking speed, and low physical activity. Individuals meeting three or more criteria are classified as frail, while one or two indicate prefrailty [11]. The FRAIL scale, a self-reported screening tool that incorporates elements of the FP and the deficit accumulation approach, evaluates fatigue, resistance, ambulation, illness, and weight loss, categorizing individuals as robust, prefrail, or frail [12, 13].

The Eastern Cooperative Oncology Group Performance Status Scale (ECOG PS) assesses functional status by evaluating self-care, daily activities, and physical ability. While it does not directly measure frailty, its relevance in this context has been established. Baseline ECOG PS has demonstrated prognostic value for overall survival in older patients with metastatic colorectal cancer, alongside the FP and geriatric screening tools such as the Geriatric 8 and the Vulnerable Elders Survey-13 [14].

In Thailand, the Thai Frailty Index (TFI) and its modified version were developed using the cumulative deficit model and have demonstrated strong predictive value for mortality [15, 16]. However, their use in clinical settings may be limited by the time required for assessment and the need to evaluate multiple variables. Frailty, as defined by the Frailty Phenotype (FP), has also been linked to falls among community-dwelling older adults [17]. More recently, the Thai version of the FRAIL scale (T-FRAIL) was validated in preoperative settings, showing good diagnostic accuracy against the TFI, and is now recommended by the Ministry of Public Health as a screening tool for older adults [18].

While widely used, both the FP and FRAIL scale primarily address the physical aspects of frailty, overlooking its multidimensional nature. Additionally, some FP components, such as handgrip strength and gait speed, may be impractical for older adults with cognitive or physical impairments, particularly in hospitalized geriatric populations [19].

The Clinical Frailty Scale (CFS) is a widely recognized tool for frailty screening and assessment used internationally. Originally developed as a 7-point scale for the Canadian Study of Health and Aging, it demonstrated a strong correlation with the Frailty Index [20]. The current version (CFS 2.0) has expanded to a 9-point scale, categorizing individuals from robust to terminally ill, based on clinical judgment [21]. The CFS has proven valuable across diverse healthcare settings, including acute care, emergency departments, and intensive care units, where it assists in prognosis and care planning [22–25]. Moreover, during public health crises such as the COVID-19 pandemic, the CFS has been suggested as a potential tool for guiding the allocation of scarce healthcare resources [26]. Its utility has been affirmed through translations and validations in multiple languages, with increasing adoption in the Asia-Pacific region [27, 28].

The Clinical Frailty Scale Classification Tree (CFS-CT) provides a structured, algorithmic approach to frailty assessment, particularly in settings where less experienced assessors are required to evaluate frailty. By guiding users through a series of systematic questions, the tool ensures consistency and minimizes reliance on subjective judgment. A previous study demonstrated high agreement between the CFS-CT and standard CFS scoring by experienced clinicians [29].

**Fig. 1:**
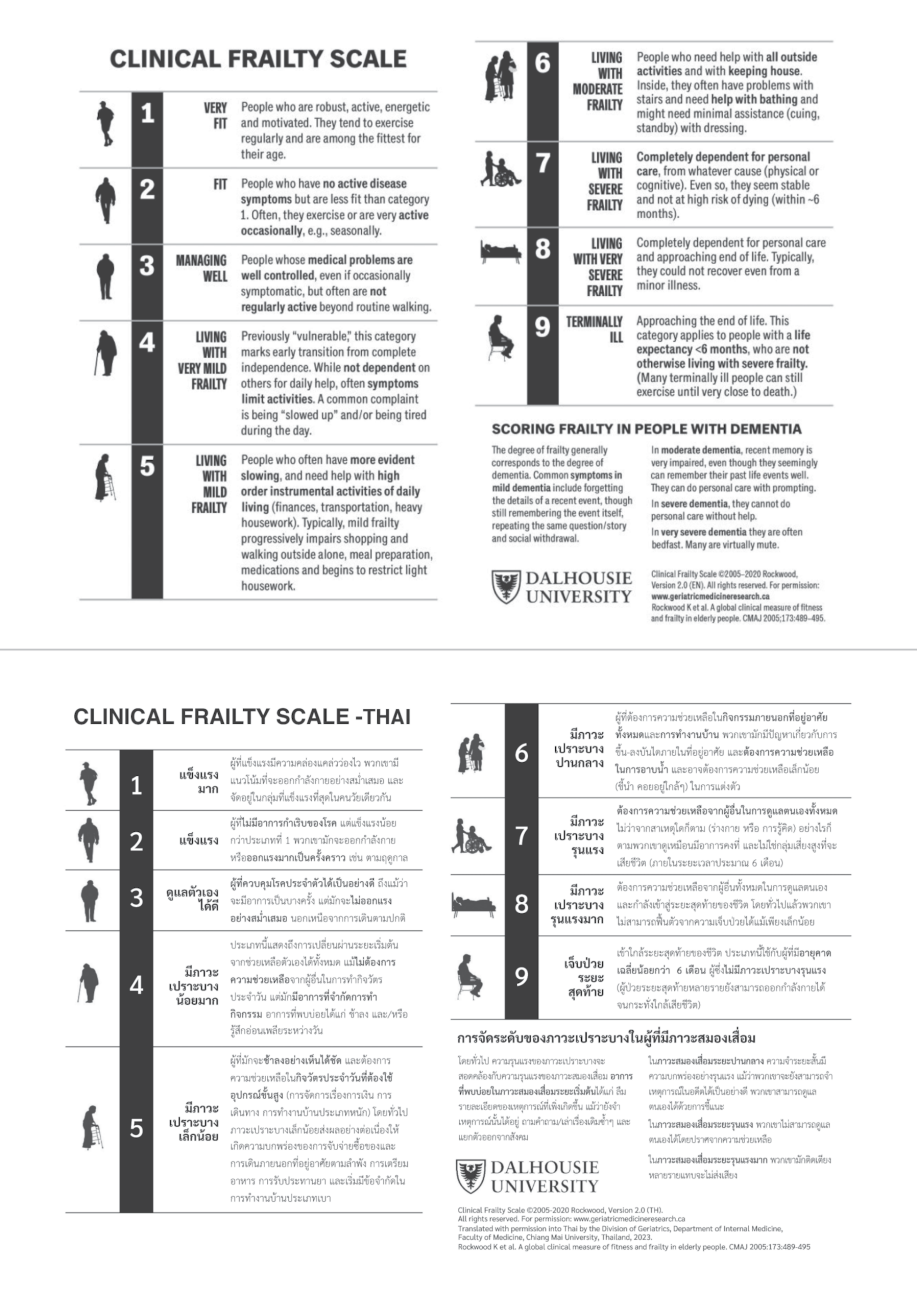
Original and Thai versions of Clinical Frailty Scale

**Fig. 2:**
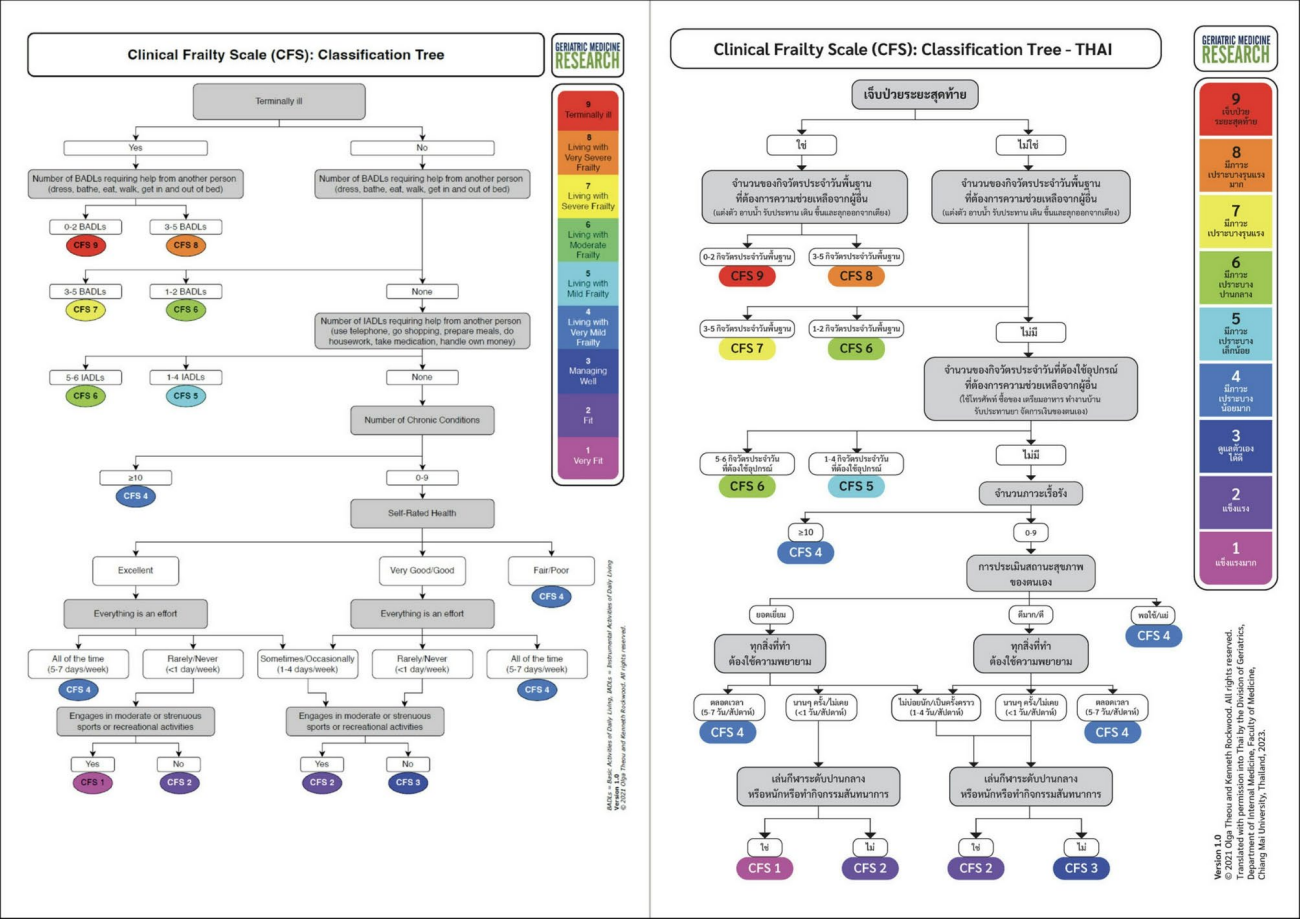
Original and Thai versions of Clinical Frailty Scale classification tree

This study aimed to provide a more comprehensive and practical frailty assessment tool by evaluating the reliability and validity of a Thai-translated version of the CFS (CFS-Thai) (Fig. 1). Additionally, it sought to assess the validity of a Thai-version of the CFS-CT (CFS-CT-Thai) (Fig. 2) in comparison to the CFS-Thai.

## Methods

This validation study was conducted in three phases at Maharaj Nakorn Chiang Mai Hospital, Faculty of Medicine, Chiang Mai University (CMU), and Bangkok Hospital Phuket (BHP), Thailand between August 2023 and June 2024. The study was approved by The Research Ethics Commitee of the Faculty of Medicine, Chiang Mai University (Approval Number: 360/2023). 

### Population

Participants were Thai nationals aged ≥ 65 years, including 127 outpatients and 86 inpatients (total *N* = 213). The exclusion criteria included dementia, altered consciousness, severe hearing impairment, unstable clinical status, and multidrug-resistant infections. All participants provided informed consent prior to enrollment.

### Process

The study was conducted in three phases [30]:

#### Phase 1: translation of the CFS and CFS-CT into Thai

With copyright permission, the CFS and CFS-CT were translated into Thai by a geriatrician and a physical therapist experienced in geriatric care. The tools were back-translated into English by a bilingual physician. An expert committee comprising two geriatricians reviewed all versions to develop pre-final Thai versions, which were pilot-tested on 20 patients. Feedback from this phase informed the final versions of the tools.

#### Phase 2: training

Two 1.5-hour training sessions were conducted to standardize assessments: one in-person at CMU and an online session for BHP staff. This ensured consistency in the use of the tools across both sites.

#### Phase 3: reliability and validity testing of CFS-Thai and CFS-CT-Thai

The reliability and validity of the CFS-Thai and CFS-CT-Thai were evaluated through inter-rater reliability and concurrent validity analyses.

### Data collection

Demographic and clinical data, including age, sex, and comorbidities, were extracted from medical records. Additional information on activities of daily living (ADLs) and self-rated health was collected using the CFS-CT-Thai.

- **Inter-Rater Reliability Testing**: Fifty-three inpatients admitted to general medical wards were independently assessed using the CFS-Thai by a geriatrician and a research nurse.
- **Concurrent Validity Testing**: All participants were assessed using the T-FRAIL, ECOG PS, and CFS-Thai. While outpatient and inpatient nurses completed evaluations using the CFS-CT-Thai on the same day.
- **Diagnostic Accuracy Testing**: In a subgroup of 61 inpatients from the CMU site, the diagnostic properties of the CFS-Thai and T-FRAIL were further evaluated against the modified Thai Frailty Index (mTFI). These participants had existing mTFI data available from another ongoing study (Approval Number: 217/2022).

### Sample size calculation

The sample size was calculated based on Chou et al., requiring a minimum of 48 participants for inter-rater reliability and 194 participants for concurrent validity [27]. Allowing for 10% attrition, the final sample size was set at 213 participants.

### Statistical analysis

Descriptive statistics were used to summarize participants’ baseline characteristics. Continuous variables were reported as means and standard deviations, while categorical variables were presented as frequencies and percentages. Inter-rater reliability of the CFS-Thai was assessed using the intraclass correlation coefficient (ICC). Agreement between the CFS-CT-Thai and the CFS-Thai was evaluated using Cohen’s kappa coefficient.

Concurrent validity was assessed using multiple comparisons. CFS-Thai and T-FRAIL scores were compared against the mTFI, which served as the reference standard, in a subset of inpatients with available data (*n* = 61), using Cohen’s kappa. Additional kappa analyses were performed using the full sample (*n* = 213) to examine agreement between the CFS-Thai and T-FRAIL, as well as between the CFS-CT-Thai and CFS-Thai. Both Spearman’s and Pearson’s correlation coefficients were calculated to evaluate the strength of association between CFS-Thai and the other frailty measures.

Sensitivity and specificity were calculated for different cut-off points of the CFS-Thai, T-FRAIL, and ECOG PS, using mTFI as the reference standard. Youden’s Index was used to determine the optimal balance between sensitivity and specificity. Diagnostic accuracy was evaluated by generating receiver operating characteristic (ROC) curves, with the area under the curve (AUC) indicating discriminatory power. Differences in AUC values between tools were compared using DeLong’s test for paired ROC curves.

All statistical analyses were conducted using Stata Statistical Software: Release 18 (StataCorp, College Station, TX: StataCorp LLC; 2023), with the significance level set at *p* < 0.05.

## Results

### Participant characteristics

The study included 213 participants (127 outpatients and 86 inpatients), with a mean age of 73.2 years (SD 6.6), and 54.0% were female. The most prevalent comorbidities were hypertension (69.5%), diabetes mellitus (34.7%), and chronic kidney disease (29.6%). Assessment of functional status revealed that the majority of participants (92.0%) had minimal impairment in basic activities of daily living (BADLs), with 0–2 impaired activities. Similarly, 92.5% of participants demonstrated moderate impairment in instrumental activities of daily living (IADLs), with 1–4 impaired activities. Self-rated health status showed marked differences between care settings: 95.3% of outpatients rated their health as good to excellent, in contrast to inpatients, among whom 66.3% rated their health as poor or fair (Table 1).

**Table 1:** Baseline characteristics of study participants by setting

Characteristics	Total (*n* = 213)	Outpatient (*n* = 127)	Inpateint (*n* = 86)
**Mean age**	73.2 (6.6)	73.4 (7.3)	73.0 (5.5)
**Female**	115 (54.0)	70 (55.1)	45 (52.3)
**Comorbidity**			
Cancer	29 (13.6)	13 (10.2)	16 (18.6)
Diabetes mellitus	74 (34.7)	49 (38.6)	25 (29.1)
Hypertension	148 (69.5)	92 (72.4)	56 (65.1)
Chronic kidney disease	63 (29.6)	32 (25.2)	31 (36.1)
Myocardial infarction	27 (12.7)	18(14.2)	9 (10.5)
Stroke	34 (16.0)	22 (17.3)	12 (14.0)
**Impaired BADLs**			
0–2	196 (92.0)	116 (91.3)	80 (93.0)
3–5	17 (8.0)	11 (8.7)	6 (7.0)
**Impaired IADLs** ^**a**^			
1–4	197 (92.5)	115 (90.5)	82 (95.4)
5–6	16 (7.5)	12 (9.5)	4 (4.6)
**Self-rated health**			
Poor/fair	63 (29.6)	6 (4.7)	57 (66.3)
Good/very good	104 (48.8)	75 (59.1)	29 (33.7)
Excellent	46 (21.6)	46 (36.2)	0 (0)

Abbreviations: BADLs, Basic activities of daily living; IADLs, Instrumental activities of daily living

Values are mean (SD) or number (%)

^a^ Excluded activities that individuals never perform

### Frailty assessment

Frailty assessments using four scales revealed distinct participant distributions (Table 2). In the CFS-Thai, the predominant category was CFS 3 (34.3%), with no participants classified in categories 8 or 9. T-FRAIL assessments identified 43.2% as robust, 42.3% as pre-frail, and 14.6% as frail. ECOG PS evaluation showed that most participants (56.3%) were restricted in strenuous activity (ECOG 1), while 25.8% maintained full activity levels (ECOG 0). Among inpatients with available mTFI scores (*n* = 61), 67.2% were classified as frail (mTFI ≥ 0.25), 27.9% as pre-frail (0.10–0.25), and 4.9% as fit (< 0.10).

**Table 2:** Frailty assessments of study participants by setting

Assessment	Total (*n* = 213)	Outpatient (*n* = 127)	Inpatient (*n* = 86)
**CFS-Thai**			
1	31 (14.5)	31 (24.4)	0 (0)
2	9 (4.2)	6 (4.7)	3 (3.5)
3	73 (34.3)	51 (40.2)	22 (25.6)
4	33 (15.5)	4 (3.2)	29 (33.7)
5	39 (18.3)	16 (12.6)	23 (26.7)
6	11 (5.2)	7 (5.5)	4 (4.7)
7	17 (8.0)	12 (9.4)	5 (5.8)
**T-FRAIL**			
Robust (0)	92 (43.2)	66 (51.9)	26 (30.2)
Pre-frail (1–2)	90 (42.3)	51 (40.2)	39 (45.4)
Frail (≥ 3)	31 (14.6)	10 (7.9)	21 (24.4)
**ECOG PS**			
0	55 (25.8)	46 (36.2)	9 (10.4)
1	120 (56.3)	65 (51.2)	55 (64.0)
2	25 (11.7)	9 (7.1)	16 (18.6)
3	13 (6.1)	7 (5.5)	6 (7.0)
mTFI*			
Fit (< 0.10)	–	–	2 (3.3)
Pre-frail (0.10–0.25)	–	–	18 (29.5)
Frail (≥ 0.25)	–	–	41 (67.2)

*mTFI data were available in a subset of 61 inpatients only

Abbreviations: CFS-Thai, Thai version of Clinical Frailty Scale; T-FRAIL, Thai version of Simple Frailty Questionnaire; ECOG PS, Eastern Cooperative Oncology Group Performance Status; mTFI Modified Thai Frailty Index

Values are number (%)

### Characteristics by CFS-Thai categories

Analysis across CFS-Thai categories revealed notable demographic and clinical patterns (Table 3). Mean age showed a positive association with frailty severity, ranging from 70.8 years (SD 7.6) in CFS 1 to 79.5 years (SD 9.5) in CFS 7. Gender distribution varied by category, with male predominance in lower frailty categories shifting to female predominance in CFS 6. The burden of chronic conditions increased with frailty severity, from 2.6 conditions (SD 1.8) in CFS 1 to 4.2 (SD 1.6) in CFS 5.

**Table 3:** Characteristics of the study participants as per CFS-Thai

	1	2	3	4	5	6	7
No. of participants	31	9	73	33	39	11	17
Mean age (years)	70.8 (7.6)	73.4 (4.2)	72.2 (4.7)	71.5 (4.3)	74.7 (6.5)	77.1 (9.3)	79.5 (9.5)
Male	11 (35.5)	7 (77.8)	29 (39.7)	20 (60.6)	20 (51.3)	3 (27.3)	8 (47.1)
Female	20 (64.5)	2 (22.2)	44 (60.3)	13 (39.4)	19 (48.7)	3 (72.7)	9 (52.9)
Number of chronic conditions	2.6 (1.8)	3.2 (1.3)	3.8 (1.7)	4 (1.7)	4.2 (1.6)	3.7 (1.2)	4 (1.3)

Values are mean (SD) or number (%)

In univariate linear regression, increasing age (β = 0.08; 95% CI: 0.05–0.11; *p* < 0.001) and a higher number of chronic conditions (β = 0.23; 95% CI: 0.09–0.36; *p* = 0.001) were significantly associated with higher CFS-Thai scores. In multivariate analysis, both age (β = 0.07; 95% CI: 0.04–0.11; *p* < 0.001) and the number of chronic conditions (β = 0.17; 95% CI: 0.04–0.30; *p* = 0.009) remained independent predictors. Female sex was not significantly associated with CFS-Thai scores in either model (Table 4).

**Table 4:** Factors associated with CFS-Thai frailty scores in univariate and multivariate linear regression models

Variable	Univariate Coefficient (95% CI)	*p*-value	Multivariate Coefficient (95% CI)	*p*-value
Age	0.08 (0.05, 0.11)	0.000	0.07 (0.04, 0.11)	0.000
Female	0.13 (-0.32, 0.59)	0.561	0.10 (-0.33, 0.53)	0.649
No. of chronic conditions	0.23 (0.09, 0.36)	0.001	0.17 (0.04, 0.30)	0.009

Multivariate models were adjusted for age and sex

### Inter-rater reliability and concurrent validity

Table 5 demonstrated the reliability and validity assessments of CFS-Thai and CFS-CT-Thai. The CFS-Thai showed strong inter-rater reliability (κ = 0.80, *p* < 0.001). At the cut-off ≥ 4, it demonstrated moderate agreement with the mTFI (κ = 0.52, *p* < 0.001), while agreement was fair at ≥ 5 (κ = 0.44, *p* < 0.001). When using T-FRAIL as the reference, the CFS-Thai showed fair agreement: κ = 0.30 for ≥ 4 and κ = 0.49 for ≥ 5 (both *p* < 0.001). T-FRAIL scores of ≥ 2 and ≥ 3 showed kappa values of 0.46 and 0.30, respectively, when compared with the mTFI. Agreement between the CFS-CT-Thai and the CFS-Thai was excellent (κ = 0.94, *p* < 0.001).

**Table 5:** Reliability and validity of CFS-Thai and CFS-CT-Thai

Tests	*N*	Kappa*	*p*-value
Inter-rater reliability	48	0.80	< 0.001
Concurrent validity			
• CFS-Thai ≥ 4 vs. mTFI	61	0.52	< 0.001
• CFS-Thai ≥ 5 vs. mTFI	61	0.44	< 0.001
• T-FRAIL (≥ 2) vs. mTFI	61	0.46	< 0.001
• T-FRAIL (≥ 3) vs. mTFI	61	0.30	< 0.001
• CFS-Thai ≥ 4 vs. T-FRAIL	213	0.30	< 0.001
• CFS-Thai ≥ 5 vs. T-FRAIL	213	0.49	< 0.001
• CFS-CT-Thai vs. CFS-Thai	213	0.94	< 0.001

Abbreviations: CFS-Thai, Thai version of Clinical Frailty Scale; mTFI, Modified Thai Frailty Index; T-FRAIL, Thai version of Simple Frailty Questionnaire; CFS-CT-Thai, Thai version of Clinical Frailty Scale Classification Tree

*Kappa values are based on dichotomized classifications using cut-off thresholds and mTFI ≥ 0.25 as the reference for frailty

### Correlation and score distribution

Correlation analyses showed moderate to strong associations between the CFS-Thai and other frailty tools (Table 6). Spearman correlation coefficients were 0.53 for T-FRAIL, 0.76 for ECOG PS, and 0.73 for mTFI (all *p* < 0.001). Pearson coefficients followed a similar trend and are presented in Table 6.

**Table 6:** Distribution of T-FRAIL, ECOG PS, and mTFI scores across CFS-Thai categories and their correlation with CFS-Thai

CFS-Thai	1	2	3	4	5	6	7	Rs	Rp
**T-FRAIL**								0.53*	0.56*
Mean (SD)	0.2 (0.5)	0.1 (0.3)	0.8 (0.8)	0.9 (0.8)	1.8 (1.4)	2.2 (0.9)	2.5 (1.5)		
Median (IQR)	0 (0, 0)	0 (0, 0)	1 (0, 1)	1 (0, 1)	2 (0, 3)	2 (1, 3)	3 (2, 3)		
**ECOG PS**								0.76*	0.78*
Mean (SD)	0.1 (0.3)	0.3 (0.5)	0.7 (0.5)	1.0 (0.2)	1.3 (0.5)	1.9 (0.9)	2.5 (0.7)		
Median (IQR)	0 (0, 0)	0 (0, 1)	1 (0, 1)	1 (1, 1)	1 (1, 2)	2 (2, 2)	3 (2, 3)		
**mTFI†**									
Mean (SD)	–	0.12 (0.11)	0.20 (0.08)	0.27 (0.07)	0.36 (0.08)	–	0.49 (0.11)	0.73*	0.75*
Median (IQR)	–	0.17 (0.00, 0.20)	0.17 (0.17, 0.27)	0.27 (0.23, 0.30)	0.35 (0.30, 0.40)	–	0.43 (0.43, 0.50)		

Abbreviations: CFS-Thai, Thai version of Clinical Frailty Scale; T-FRAIL, Thai version of Simple Frailty Questionnaire; ECOG PS, Eastern Cooperative Oncology Group Performance Status; mTFI Modified Thai Frailty Index

Values are presented as mean (SD) and median (IQR)

† mTFI data were available for a subset of 61 inpatients

R_s_ and R_p_ were calculated using Spearman correlation and Pearson correlation, respectively

*p < 0.001

All three scales showed consistent progression across CFS-Thai categories. For T-FRAIL, mean scores increased from 0.2 (SD 0.5) in CFS 1 to 2.5 (SD 1.5) in CFS 7. ECOG PS scores rose from 0.1 (SD 0.3) in CFS 1 to 2.5 (SD 0.7) in CFS 7. Among inpatients with available mTFI data (*n* = 61), the mean mTFI score increased from 0.12 in CFS 2 to 0.49 in CFS 7, reflecting increasing frailty severity across CFS-Thai categories.

### Diagnostic performance

Diagnostic performance was assessed for each tool using mTFI as the reference standard (Table 7). For T-FRAIL, a cut-off point of ≥ 2 yielded a sensitivity of 70.7% and specificity of 80.0% (AUC = 0.75, 95% CI: 0.64–0.87, *p* = 0.001). At a higher cut-off point of ≥ 3, sensitivity dropped to 39.0%, while specificity reached 100.0%; the AUC remained acceptable at 0.70 (95% CI: 0.62–0.77, *p* = 0.025), despite perfect specificity.

**Table 7:** Diagnostic properties of T-FRAIL, CFS-Thai and ECOG PS for frailty detection using mTFI as the reference standard

Tool	Cutoff-points	Sensitivity % (95% CI)	Specificity % (95% CI)	AUC (95% CI)	Youden Index	*p*-value
T-FRAIL	≥ 2	70.7 (54.5–83.9)	80.0 (56.3–94.3)	0.75 (0.64–0.87)	50.7	0.001
T-FRAIL	≥ 3	39.0 (24.2–55.5)	100.0 (83.2–100.0)	0.70 (0.62–0.77)	39.0	0.025
CFS-Thai	≥ 4	92.7 (80.1–98.5)	55.0 (31.5–76.9)	0.74 (0.62–0.86)	47.7	< 0.001
CFS-Thai	≥ 5	93.5 (79.3–98.2)	79.3 (61.6–90.2)	0.86 (0.81–0.92)	53.5	< 0.001
ECOG PS	≥ 2	83.9 (67.4–92.9)	93.3 (78.7–98.2)	0.89 (0.82–0.95)	42.5	0.001
ECOG PS	≥ 3	35.5 (21.1–53.1)	96.7 (83.3–99.4)	0.67 (0.59–0.76)	12.5	< 0.001

*All diagnostic performance metrics were calculated in a subset of 61 inpatients with available mTFI scores

Abbreviations: AUC, Area Under the Curve; CI, Confidence Interval; T-FRAIL, Thai version of Simple Frailty Questionnaire; CFS-Thai, Thai version of Clinical Frailty Scale; ECOG PS, Eastern Cooperative Oncology Group Performance Status; mTFI, Modified Thai Frailty Index

The CFS-Thai demonstrated robust diagnostic performance. At the ≥ 4 cut-off, it yielded a sensitivity of 92.7% and specificity of 55.0% (AUC = 0.74, 95% CI: 0.62–0.86, *p* < 0.001). Increasing the threshold to ≥ 5 maintained high sensitivity (93.5%) while improving specificity to 79.3% (AUC = 0.86, 95% CI: 0.81–0.92, *p* < 0.001).

**Fig. 3:**
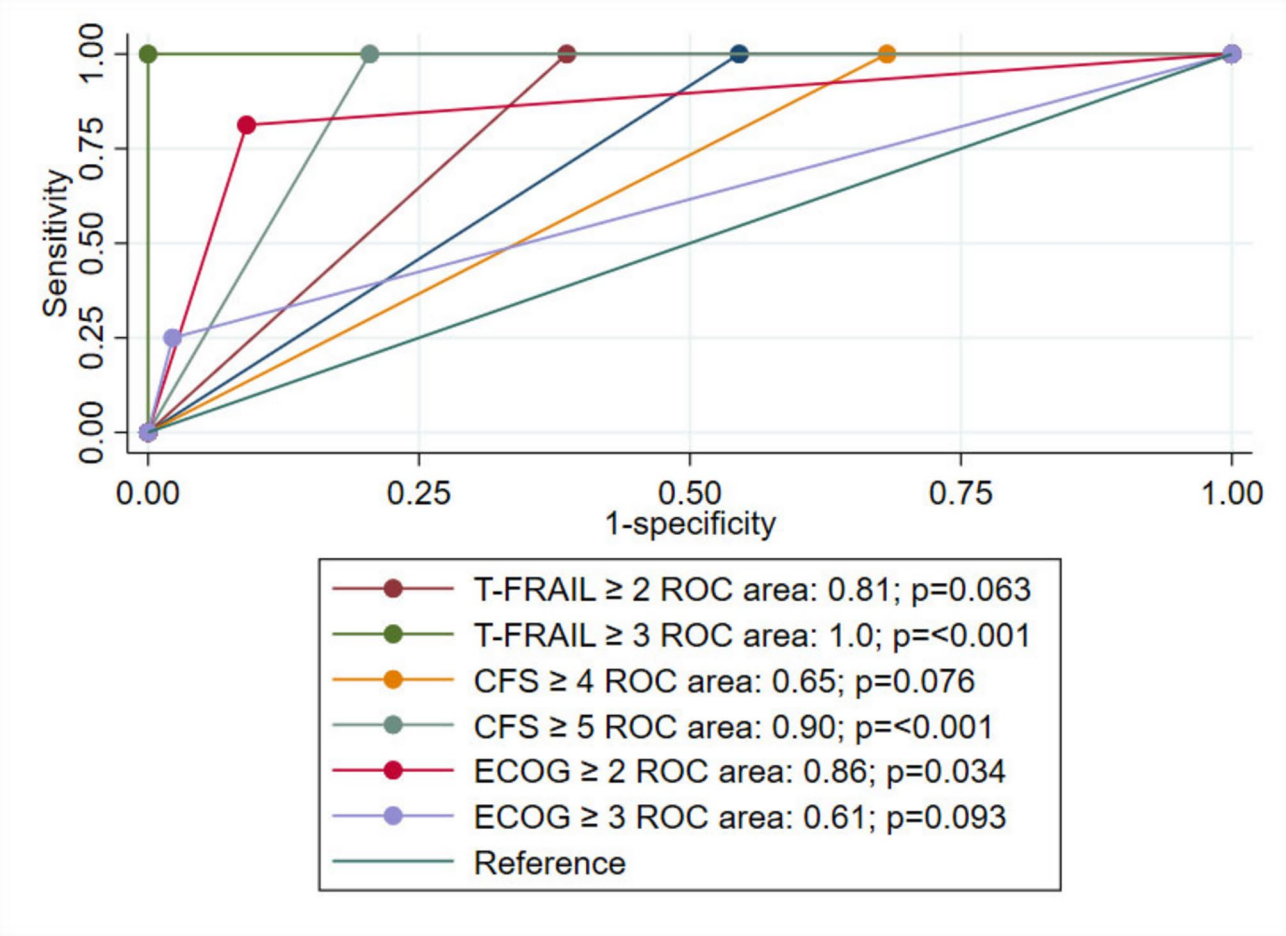
AUC of ROC for different cut-off points of the T-FRAIL, CFS-Thai, and ECOG PS using mTFI as the reference standard

ECOG PS also showed strong diagnostic accuracy. At a cut-off point of ≥ 2, sensitivity was 83.9% and specificity 93.3% (AUC = 0.89, 95% CI: 0.82–0.95, *p* = 0.001). Increasing the cut-off to ≥ 3 improved specificity to 96.7% but reduced sensitivity to 35.5% (AUC = 0.67, 95% CI: 0.59–0.76, *p* < 0.001). Figure 3 presents ROC curves for multiple cut-off points of the CFS-Thai, T-FRAIL, and ECOG PS, using mTFI as the reference standard. We performed DeLong’s test to compare the AUCs of the three most clinically relevant thresholds: CFS-Thai (≥ 5), T-FRAIL (≥ 2), and ECOG PS (≥ 2). No statistically significant differences were observed between any of these pairwise comparisons (*p* > 0.05).

## Discussion

The CFS-Thai demonstrated robust diagnostic properties. The CFS-Thai demonstrated strong inter-rater reliability (κ = 0.80), exceeding that of the Chinese version (κ = 0.60) [26]. This may reflect the clinical expertise of our assessors, particularly the involvement of a geriatrician and a research nurse in frailty classification.

We evaluated the concurrent validity of the CFS-Thai by comparing it with T-FRAIL, ECOG PS, and mTFI. This included both categorical agreement (Cohen’s kappa) and continuous correlation (Spearman and Pearson coefficients). For a subset of inpatients, we used the mTFI as the reference standard.

Kappa values between the CFS-Thai and the mTFI indicated moderate agreement at the cut-off point ≥ 4 (κ = 0.52) and fair agreement at ≥ 5 (κ = 0.44), suggesting modest diagnostic consistency between the tools. Although both instruments are based on the cumulative deficit model, differences in tool structure and item composition may explain the limited agreement. The original Canadian study reported a strong correlation (ρ = 0.80) between the CFS and the 70-item Frailty Index [20], but that analysis used a different statistical method and a more comprehensive reference standard.

One likely explanation for the observed discrepancy lies in structural differences between the mTFI and the original 70-item Frailty Index. While the original index encompasses a broad spectrum of deficits with multiple items per domain, the mTFI uses a condensed set of 30 items, typically representing each domain with only one or a few indicators. This limited item distribution may reduce domain coverage and contribute to the lower agreement observed with the CFS-Thai, which incorporates broader clinical judgment across multidimensional health domains. Additionally, the use of a limited inpatient-only subsample for mTFI analysis (*n* = 61) may have further influenced the observed level of agreement.

Our correlation analyses further supported the validity of the CFS-Thai. It showed moderate correlation with T-FRAIL (Spearman’s correlation coefficient, ρ = 0.53) and strong correlation with ECOG PS (ρ = 0.76) and mTFI (ρ = 0.73). The simpler structure of ECOG PS may introduce less measurement variability, which could also contribute to its stronger association with the CFS-Thai. This pattern indicates moderate alignment with symptom-based assessments and stronger alignment with ECOG PS, a clinician-rated tool focused on functional status, and with mTFI, which shares conceptual overlap in domains such as comorbidity, physical function, and activities of daily living.

To place our correlation results in a regional context, we compared them with those reported in the Korean validation study. The correlation between the CFS-Thai and other frailty measures was slightly lower in our sample. In our study, Spearman’s correlation coefficients were ρ = 0.53 for T-FRAIL and ρ = 0.76 for ECOG PS, whereas the Korean study reported ρ = 0.80 for K-FRAIL and ρ = 0.92 for ECOG PS [25]. These differences likely reflect variations in study populations. The Korean cohort consisted primarily of older, frailer inpatients, whereas our sample was younger, predominantly outpatient-based, and concentrated in CFS categories 1–4, with no representation in categories 8 or 9. The high proportion of participants with low ECOG PS scores in our study may have contributed to greater variability in frailty status, potentially attenuating correlation coefficients.

Mean T-FRAIL, ECOG PS, and mTFI scores progressively increased across higher CFS-Thai categories, supporting the scale’s ability to differentiate between varying levels of frailty severity. For example, T-FRAIL scores rose from a mean of 0.2 in CFS 1 to 2.5 in CFS 7; similarly, ECOG PS increased from 0.1 to 2.5, and mTFI from 0.12 to 0.49. This gradient in scores supports the discriminant validity of the CFS-Thai, reflecting its capacity to distinguish among robust, pre-frail, and frail individuals. While mTFI findings support the concurrent and diagnostic validity of the CFS-Thai, these results were derived from a smaller inpatient subsample and should be interpreted cautiously. This pattern of score progression also reinforces the known-groups validity of the CFS-Thai and its consistency with other established frailty measures.

The CFS-Thai demonstrated strong diagnostic performance when evaluated against the mTFI. A cut-off of ≥ 4 yielded high sensitivity (92.7%) but lower specificity (55.0%), supporting its utility as a screening tool. Increasing the threshold to ≥ 5 improved specificity to 79.3% while maintaining high sensitivity (93.5%). At this cut-off, the CFS-Thai achieved the highest Youden Index (53.5) and an AUC of 0.86 (95% CI: 0.81–0.92), surpassing the performance observed at the ≥ 4 threshold. This result is consistent with findings from the Chinese validation study, which reported that raising the CFS cut-off improved specificity without significantly reducing sensitivity [25].

ECOG PS also showed strong diagnostic accuracy, with a cut-off point of ≥ 2 yielding balanced sensitivity (83.9%) and specificity (93.3%) and the highest AUC (0.89, 95% CI: 0.82–0.95) and Youden Index (77.2) among all tools tested. Raising the threshold cut-off point to ≥ 3 significantly improved specificity (96.7%) but reduced sensitivity (35.5%), aligning with previous findings in the Korean study where ECOG PS ≥ 3 was associated with more severe frailty [26].

Our study has several strengths. We included both outpatient and inpatient older adults from two tertiary care hospitals, allowing for clinical diversity across care settings. While this enhances internal consistency in assessment and staff training, it may limit external generalizability. Additionally, we conducted a comprehensive comparison of the CFS-Thai and CFS-CT-Thai with established frailty and functional assessment tools, using multiple analytic approaches, including correlation, agreement, and diagnostic performance metrics.

Several limitations should be acknowledged. First, the exclusion of participants with dementia, altered consciousness, or severe frailty (CFS categories 8–9) limits the applicability of our findings to more vulnerable or functionally impaired populations. Second, although both study sites offered varied clinical services, their status as tertiary care centers may not fully represent frailty profiles in rural, community-based, or primary care settings. Third, we did not investigate associations between CFS-Thai scores and key clinical outcomes such as hospital length of stay, readmission, or mortality, which limits conclusions about its prognostic value. Fourth, subgroup or sensitivity analyses across clinical settings (e.g., outpatient vs. inpatient) or frailty severity levels were not conducted, consistent with the scope and sample size of this validation-focused study. Analyses involving the modified Thai Frailty Index (mTFI) were limited to an inpatient-only subsample (*n* = 61). While these constraints are typical for early validation work, they may limit insights into potential performance differences across subpopulations. Finally, the analyses did not adjust for potential confounders such as age, sex, or comorbidity burden, which may have influenced observed associations.

Future research should address these limitations by enrolling more diverse populations from different healthcare settings, including cognitively impaired and severely frail individuals. Incorporating multivariable models and longitudinal outcome tracking will further clarify the clinical utility and generalizability of the CFS-Thai and CFS-CT-Thai, thereby informing their potential integration into national screening protocols and routine geriatric care.

## Conclusion

The CFS-Thai and CFS-CT-Thai demonstrated strong reliability and acceptable validity for frailty assessment in a clinically diverse sample of Thai older adults. The high level of agreement between them supports their use in clinical settings, particularly where geriatric expertise is limited and simpler tools like the classification tree may offer practical advantages.

However, broader applicability, especially in rural or community-based contexts and among cognitively impaired or severely frail populations, requires further investigation.

Future research should evaluate these tools in broader healthcare contexts, assess their predictive value for outcomes such as hospitalization and mortality, and evaluate their influence on clinical decision-making and resource allocation. These steps will be essential to support responsible adoption within Thailand’s national strategy for healthy aging.

## Data Availability

The data that support the findings of this study are available from the corresponding author, PJ, panas.j@cmu.ac.th upon reasonable request.

## Abbreviations

CFS: Clinical Frailty Scale
CFS-CT: Clinical Frailty Scale Classification Tree
CFS-Thai: Thai version of CFS
CFS-CT-Thai: Thai version of CFS-CT
FRAIL scale: The Simple Frailty Questionnaire
T-FRAIL: Thai version of the FRAIL scale
ECOG PS: Eastern Cooperative Oncology Group Performance Status
FP: Frailty Phenotype
mTFI: Modified Thai Frailty Index
CMU: Maharaj Nakorn Chiang Mai Hospital, Faculty of Medicine, Chiang Mai University
BHP: Bangkok Hospital Phuket
ICC: Intraclass Correlation Coefficient
ROC: Receiver Operating Characteristic
AUC: Area Under the Curve
SD: Standard Deviation
IQR: Interquartile Range
